# Supplementary material for: The interactome of intact mitochondria by cross-linking mass spectrometry provides evidence for coexisting respiratory supercomplexes
Source: Mol Cell Proteomics. 2017 Dec 8;17(2):216–32. doi: 10.1074/mcp.RA117.000470 (PMC5795388; doi:10.1074/mcp.RA117.000470)
Supplement: Supplemental Data [file supp_RA117.000470_133922_0_supp_23978_fzffwf.zip › spectra_annotation/mito_spectra_annotation/283-1-1-1-8-1.pdf]

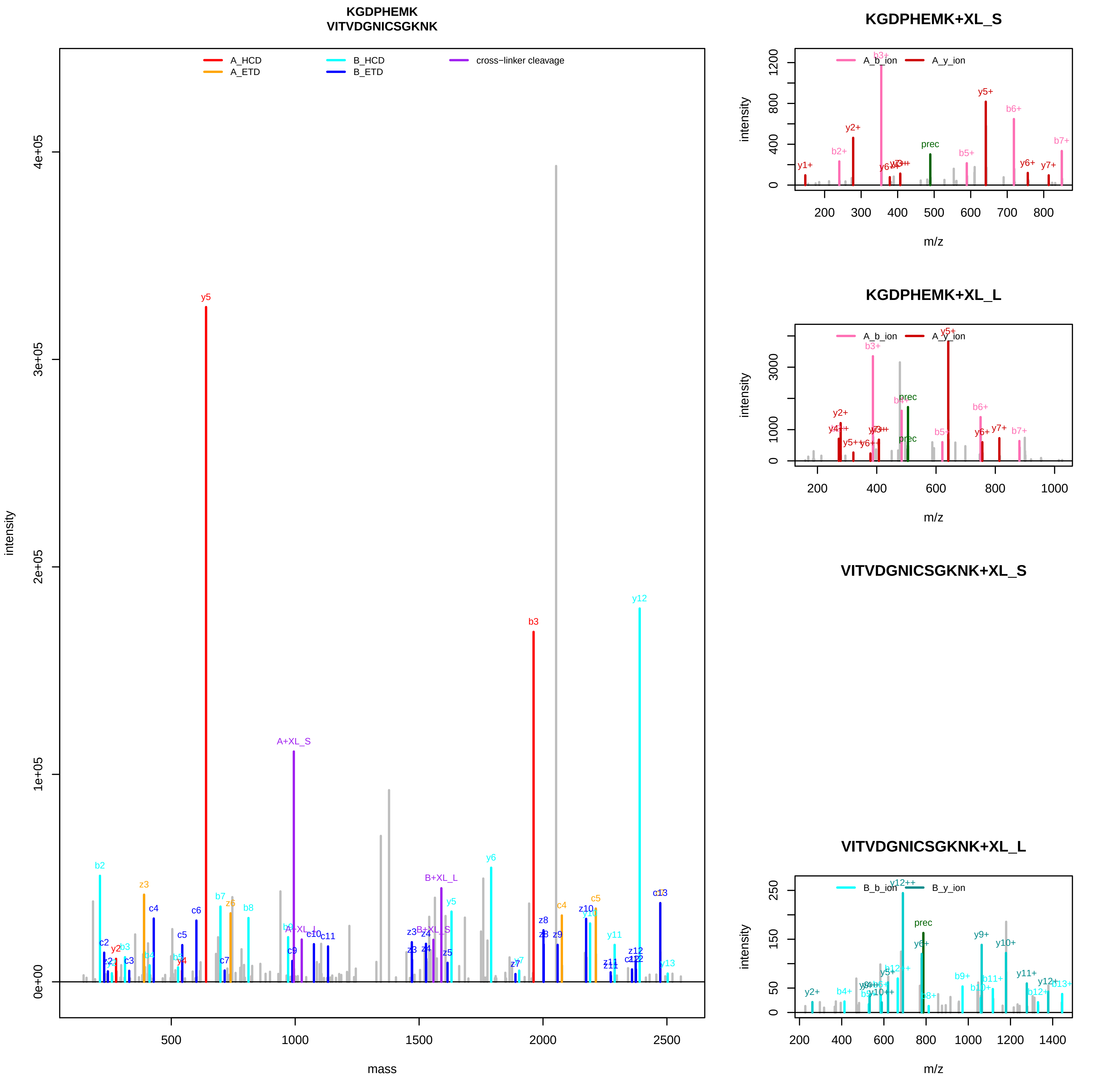
KGDPHEMK
VITVDGNICSGKNK
A_HCD
A_ETD
B_HCD
B_ETD
cross−linker cleavage
intensity
mass
0e+00
1e+05
2e+05
3e+05
4e+05
500
1000
1500
2000
2500
y5
b3
y12
A+XL_S
B+XL_L
b2
z3
c4
c6
c5
b7
z6
b8
c2
y2
b3
c3
c7
y6
y5
z8
z9
c4
z10
c5
y11
z12
c12
c13
y13
c9
c10
c11
z3
z4
z5
z7
KGDPHEMK+XL_S
A_b_ion
A_y_ion
intensity
m/z
0
400
800
1200
200
300
400
500
600
700
800
b3+
y5+
b6+
y2+
prec
b2+
b5+
b7+
y1+
y6+
y7+
KGDPHEMK+XL_L
0
1000
3000
200
400
600
800
1000
y5+
b3+
prec
b4+
b6+
y2+
b5+
y6+
y7+
b7+
VITVDGNICSGKNK+XL_S
VITVDGNICSGKNK+XL_L
B_b_ion
B_y_ion
0
50
150
250
200
400
600
800
1000
1200
1400
y12++
prec
y6+
y9+
y10+
y11+
b9+
b11+
y12+
y2+
b4+
b13+
